# Biomediators in Polycystic Ovary Syndrome and Cardiovascular Risk

**DOI:** 10.3390/biom11091350

**Published:** 2021-09-12

**Authors:** Srdan Pandurevic, Djuro Macut, Flaminia Fanelli, Uberto Pagotto, Alessandra Gambineri

**Affiliations:** 1 Unit of Endocrinology and Diabetes Prevention and Care, IRCCS Azienda Ospedaliero-Universitaria di Bologna, University of Bologna, 40138 Bologna, Italy; srdan.panurevic@unibo.it (S.P.); flaminia.fanelli2@unibo.it (F.F.); uberto.pagotto@unibo.it (U.P.); 2 Clinic of Endocrinology, Diabetes and Metabolic Diseases, Faculty of Medicine, University of Belgrade, 11000 Belgrade, Serbia; djmacut@gmail.com

**Keywords:** PCOS, cardiovascular risk, biomediators, insulin resistance, sex steroids, SHBG

## Abstract

Polycystic ovary syndrome (PCOS) is extremely heterogeneous in terms of clinical manifestations. The variability of the syndrome’s phenotype is derived from the genetic and molecular heterogeneity, with a great deal of environmental factors that may have long-term health consequences, such as metabolic and cardiovascular (CV) diseases. There is no doubt that women with PCOS suffer from metabolic complications more than their age-matched counterparts in the general population and at an earlier age. Obesity, low steroid hormone-binding globulin (SHBG), hyperandrogenemia, insulin resistance, and compensatory hyperinsulinemia are biomediators and early predictors of metabolic complications in PCOS. Doubts remain about the real risk of CV diseases in PCOS and the molecular mechanisms at the basis of CV complications. Based on that assumption, this review will present the available evidence on the potential implications of some biomediators, in particular, hyperandrogenism, estrogen-progesterone imbalance, insulin resistance, and low SHBG, in the processes leading to CV disease in PCOS, with the final aim to propose a more accurate CV risk assessment.

## 1. Introduction

Polycystic ovary syndrome (PCOS) is one of the most common endocrinological diagnoses in women, even though there is great variance in the prevalence reports. Depending on the source, the number is between 4% and 18%, with the main difference between the sources being the diagnostic criteria; this demonstrates the notable clinical heterogeneity of the syndrome [1]. In an effort to achieve a more precise diagnosis and promote comparable research in the field, the latest European Society of Human Reproduction and Embryology (ESHRE) criteria covered four different combinations of signs and symptoms (clinical or biochemical hyperandrogenism, oligo-anovulation, polycystic ovarian morphology at ultrasound), giving four phenotypically diverse groups, which are probably genotypically diverse as well [2]. Earlier research into genetics of PCOS provided few insights [3], but this area has been recently investigated using genome-wide association studies (GWAS), and some distinct patterns have emerged. These analyses identified PCOS-specific polymorphisms in the genes regulating gonadotropin activity, androgen biosynthesis, and caloric homeostasis [4,5], which goes in line with some of the proposed pathophysiological mechanisms. There were also attempts to go a step further and explain the variability of the phenotype using unsupervised cluster analysis, with a goal of connecting subgroup specifics to genome polymorphisms. One study explored GWAS data using this method with eight different clinical parameters—they identified two distinct subgroups, dubbed “reproductive” and “metabolic”, based on differences in body mass index (BMI), luteinizing hormone (LH), sex hormone-binding globulin (SHBG), and fasting insulinemia and glycemia [6]. This genetic heterogeneity could be the basis of the variability of syndrome’s phenotype, with a great deal of environmental factors on top, influencing the long-term consequences of PCOS. There is no doubt that women with PCOS suffer from metabolic complications more than their age-matched counterparts in the general population and at an earlier age, as well as having obesity, low SHBG, hyperandrogenemia, insulin resistance (IR), and hyperinsulinemia that are risk factors for, and early predictors of, metabolic complications in PCOS [7,8,9]. Since metabolic complications are strongly correlated with cardiovascular (CV) disease, this raises the question of whether PCOS really carries an inherent risk of CV morbidity. There is no satisfying answer for now based on scarce and contradictory available data. This review will describe the biomediators that are shown to be altered in PCOS and potentially implicated in the processes leading to CV disease.

## 2. Atherosclerosis and Cardiovascular Disease

CV disease still mounts the greatest burden on life expectancy worldwide, at 31.4% of all mortality; 90% of that number is caused by atherosclerotic disease [10]. Atherosclerosis is a relentless, continuous process of blood vessel thickening that causes death by finally occluding cerebral or coronary arteries. The process of atherosclerosis has been explained in the last decades, but with important details at the start of the process and final occlusion events left poorly understood [11]. We are briefly going to provide a basic description of the process, concentrating on the input variables that affect the speed of the progression of atherosclerosis in critical points and are known to be altered in PCOS patients.

The precursors of atherosclerosis, fatty streaks, have a predilection for arterial walls with low blood flow shear stress [12]. Low wall shear stress causes a local increase in concentration of macromolecules (like lipoproteins), but also a reduction in oxygen transport efficiency. Local hypoxia causes the production of reactive oxygen species (ROS) in mitochondria by electron leakage from the respiratory chain [13]. This process by itself does not explain the deposition of lipids in the arterial intima, however. Lipids, being hydrophobic, cannot dissolve in blood, so they are transported through the body in two main ways: erythrocytes and lipoproteins [14]. Of lipoproteins, it has been recognized that those containing the apo-B100 apolipoprotein are the ones mainly deploying lipids in arterial walls [15]. Deployment of lipids happens in the aforementioned favorable sites when there is a dysfunction of endothelial cells. Arterial endothelium is very effective at resisting this infiltration in optimal conditions, using several mechanisms. Firstly, healthy endothelium is smooth and without gaps between cells [16]. Through the MEK5/ERK5/MEF2 pathway [17,18], a constitutional activity of endothelial nitric oxide synthase (eNOS) is achieved, which in turn stimulates endothelial cell migration and maintains the integrity of the endothelial barrier [19]. Nitric oxide (NO), apart from its vasodilatory effect, also inhibits the nuclear factor kappa B (NF-κB), preventing inflammatory response and endothelial activation [20]. Damage by oxidative stress is controlled by superoxide dismutase (SOD), which neutralizes ROS [21]. There also exists a reactive protective pathway, in cases of transient hypoxia, that is regulated by hypoxia-inducible factor 1 (HIF1) [22]. The HIF1 pathway was a Nobel-worthy discovery, being a common endpoint for various biomediators. Classically upregulated by hypoxia, HIF1 activity is also regulated by many different growth factors, with the major ones being inflammatory cytokines and insulin, through the mechanistic target of the rapamycin (mTOR) pathway. HIF1 also upregulates vascular endothelial growth factor (VEGF), and through it, angiogenesis as well [23].

The breakdown of these systems can happen in a number of ways. Lipoproteins, being micellar in structure, continuously diffuse in and out through the endothelium of the intimal space depending on their concentration and the blood Reynolds number adjacent to the arterial wall. In the environment of local hypoxia, increased concentration, and unregulated oxidative stress, these particles enter the intimal space, get bound to the proteoglycans of the matrix, and get oxidized by the ROS [24]. High oxidative stress also overpowers the inhibitory mechanisms for NF-κB, which gets activated and promotes endothelial activation [16,25]. Presence of advanced glycosylation end products (AGEs) and oxidized low-density lipoprotein (LDL) also favor endothelial activation. Activated endothelium is porous and expresses various inflammatory and adherence proteins. Passing monocytes are attracted by these chemotactic factors and transform into macrophages once they enter the intima. Macrophages and dendritic cells phagocytize oxidized lipids, eventually becoming foam cells and depositing eaten lipids as fatty streaks, a process that will lead to formation of the atherosclerotic plaque [26].

In PCOS, alterations have been described in many of the aforementioned mechanisms [27,28,29,30]. However, it is important to differentiate the effects of specific, known hormonal biomediators from the possibility of PCOS being intrinsically predisposed for atherosclerosis, of which there is scarce evidence. There are a few examples of this. The HIF-1 pathway, and consequently VEGF, has been shown to be downregulated in PCOS, which has consequences not only for CV risk, but fertility as well, since this pathway is integral to endometrial function [31]. In this case, further analysis of mRNA expression showed that body weight was a much bigger factor than PCOS in influencing HIF-1 and VEGF activity [32]. One of the disorders connected to PCOS is low-grade inflammation, itself directly implicated in systemic endothelial dysfunction; low-grade inflammation was shown to be significantly reduced when PCOS patients were treated with metformin, an insulin-sensitizing drug, thus suggesting that insulin resistance is one of the biomediators of low-grade inflammation in PCOS [33].

With consideration of those findings, together with the inconclusiveness of longitudinal studies on CV risk [34], one reasonable assumption would be that the diagnosis of PCOS per se does not signify an increased CV risk, but rather represents the condition with existing CV risk factors that needs further evaluation, which would provide a more accurate CV risk assessment. Based on that assumption, the following sections will present the available evidence on the potential implication of some biomediators, in particular hyperandrogenism, estrogen-progesterone imbalance, insulin-resistance, and low SHBG, in the processes leading to CV disease in PCOS.

## 3. Potential Biomediators of Cardiovascular Risk in PCOS

### 3.1. Hyperandrogenism

The current consensus is that elevated androgens in women, on the order of magnitude seen in PCOS, are linked with elevated CV risk markers [35,36,37,38], but the data is much more ambiguous when evaluating their relation to CV morbidity and death [39,40].

Main active androgens, i.e., androgens able to bind to androgen receptor (AR), are testosterone (T), 5α-dihydrotestosterone (5α-DHT), and androstenedione (4-A). 4-A is a weak AR agonist but also a precursor, through 17β-HSD, for T and for 5α-DHT. Very recently, other active androgens have been described—the 11-oxygenated androgens (11-keto-T, 11-keto-DHT, and others), but with unclear clinical significance [41].

There exists one type of AR, and its main activity is that of a transcription modulator as it gets dimerized (by ligand binding) and transported into the cellular nucleus. ARs are present in various tissues in body, exhibiting diverse effects, and its effect on vascular smooth muscle cells (VSMC) is especially heterogeneous. One pathway includes AR-activated transcription of Alpl mRNA, a marker of cellular calcification. Conversely, AR also binds to the androgen-response element in the promotor of the growth arrest-specific gene 6 (Gas6), an antiapoptotic agent, which, through the phosphoinositide 3-kinase (PI3K)/protein kinase B (Akt) mechanism, inhibits phosphate-induced calcification of VSMC [42]. Additionally, AR induces transcription of the ADTRP (androgen-dependent tissue factor pathway inhibitor regulating protein) gene, whose ADTRP protein both induces transcription and augments activity of the tissue factor pathway inhibitor (TFPI) [43]. TFPI finally inhibits the generation of the tissue factor Xa by factor VIIa in the coagulation pathway [44].

There is also increasing evidence of short-acting effects by AR, hypothesized to come from membrane complexes of AR coupled to G proteins, and possibly other mechanisms. Physiologically, it is improbable to have a transcriptional effect that is measured in minutes, so these effects are regarded as nongenomic. AR is shown to non-genomically mediate phosphorylation of eNOS by binding directly to PI3K/Akt, thus increasing NO production in the endothelium [45]. Additionally, in male mice osteoblasts, parenteral T causes a rapid (5–10 s delay) increase in intracellular calcium ion concentration by activating the pertussis toxin-sensitive G protein-phospholipase C (PLC)/inositol 1,4,5-trisphosphate (IP3) signaling pathway. This activity was not blocked by AR antagonists, allowing for a possibility of a novel membrane binding site for non-transcriptional androgen effects [46]. Currently no suitable candidate membrane receptor has been recognized that could explain all the aforementioned nongenomic effects. Interestingly, the same experimental protocol failed to produce such an effect in female mice osteoblasts, thus suggesting a sex-related effect. Few other nongenomic pathways are hypothesized; a major example is the activation of members of the mitogen-activated protein kinase (MAPK) family, Raf-1 and ERK-2, through membrane signaling, which regulates Ca^2+^ cytoplasmic concentration and eNOS activity [47]. Ca^2+^ ion concentration is of course pertinent to many pathways; in this case, a hypothesis can be made of it mediating T’s nongenomic effect on VSMC contraction state (tone) and endothelial activation.

Therefore, in vivo activity of androgens is a mix of genomic and nongenomic effects, but also the consequence of the impact of androgens on the metabolic milieu. Insulin resistance and increase of visceral fat are the most well-understood negative effects of hyperandrogenism in women, which in turn are implicated in maintaining hyperandrogenism and in increasing CV risk also through the impact on chronic low-grade inflammation [27].

T is the main active circulating androgen, and it is classically used to diagnose hyperandrogenism in women with PCOS. However, current state-of-the-art hyperandrogenism in PCOS is expanded to DHT, 4-A, formulaic estimations of free T [48], as well as 11-oxygenated androgens. 4-A in particular is the most frequently increased androgen in PCOS [49]. Moreover, it has been recently demonstrated that 11 oxygenated androgens make up more than 50% of circulating androgens in PCOS [41].

Therefore, PCOS is characterized by hyperandrogenism, which by itself and through different mechanisms can increase risk of CV disease. Nevertheless, in PCOS there is a need for a more precise definition and quantification of hyperandrogenism [50], with an aim to provide more reliable tools to evaluate future CV disease risk.

### 3.2. Estrogen-Progesterone Imbalance

In healthy pre-menopausal women, estradiol (E2) is the most potent circulating estrogen, and most of E2 comes from the activity of aromatase in granulosa cells of the ovary by conversion of thecal T. This makes E2 very dependent on cyclical changes in the ovary during the menstrual cycle, and also on age-related transformations. It was shown that average E2 levels in women are stable or slightly increase during the reproductive years, and then around two years before menopause, they start to fluctuate wildly and then practically disappear from the circulation with menopause. This coincides with the aforementioned increase in CV disease in women. This association sparked questions about the potential beneficial effect of hormonal replacement therapy (HRT) in reducing CV events in women in the menopausal period. However, no clinical trial to date has shown a benefit through either CV disease risk makers like carotid intima media thickness (cIMT) or CV events incidence. The Women’s Health Initiative study (WHI) registered increased CV events in women who took HRT in post-menopause, but that study had a limitation in that the women were already 10 or more years post-menopausal when they started the trial [51]. A recently published RCT (Kronos Early Estrogen Prevention Study), performed in early-state menopausal women without pre-existing CV disease, has not shown an increase in CV events nor in CVD risk markers (cIMT, coronary artery calcium), but did not show a benefit to HRT in this regard either [52].

One of the earliest changes in vascular function associated with the development of atherosclerosis and, therefore, with CV diseases is the loss of endothelium-derived NO. The endothelial NO production is stimulated by estrogens [53], and this effect can be slow-acting, sustained, and therefore probably genomic, or it can be quick, short-acting, and nongenomic [54]. Nongenomic activity of estrogens was discovered before androgen-mediated nongenomic pathways and it is better studied; it was shown that E2 binds to a specific G-protein-coupled membrane receptor, GPR30, activating rapid signaling pathways PI3K/Akt and mitogen-activated protein kinase (MAPK) [55]. Downstream, it is shown that acute administration of E2 causes vasodilation by stimulating the production of NO in VSMC [56].

The genomic effects of estrogens are initiated by their binding to the estrogen receptors (ERs). Estrogens are ligands for two different cytoplasmic receptors: ER-α and ER-β. They are highly homologous, but they regulate gene expression differently. Different experiments have shown various combinations of ER-α- and ER-β-related effects, depending on the tissue affected. For example, several studies used the model of VSMC proliferation, and they found that even though both are protective by themselves, only when both are present is the VSMC proliferation regulated normally [53].

Interestingly, ER-α and ER-β expression also changes independently during the menstrual cycle and the occurrence of ovulation—ER-α in vascular endothelium was 30% lower in early compared to late follicular phase, and post-menopausal women also continuously had 33% lower expression than pre-menopausal women in postovulatory phase [57]. There are no data on vascular ER-β in humans specifically, but measurements in myometrium do show an increase in ER-β and decrease in ER-α in post-menopause, when compared to pre-menopause.

In PCOS there are still conflicting accounts on if and how exactly ER expression is different. Alterations of ER-α, ER-β, and GPR30 expression in ovaries and endometrium are well supported by data [58,59,60], but to our knowledge there are no studies that have investigated their expression in PCOS in non-reproductive tissues. More research is needed, but this difference in the receptor makeup could explain some differences of PCOS with regards to the general population in terms of estrogen effects on CV risk, even without major differences in circulating hormones.

There is also evidence of estrogen-progesterone interaction in the vasculature, mediated by endothelin-1 (ET-1) [61,62]. ET-1 is the most powerful endogenous vasoconstrictor. ET-1 is produced by endothelin-converting enzyme (ECE) from its precursor prepro-ET-1, and it has G protein-coupled ET type A receptors (ETAR) and ET type B receptors (ETBR) in VSMC, and ETBR in endothelium. VSMC, ETAR, and ETBR mediate vasoconstriction, while endothelial ETBR induces the production of NO and other endothelial vasodilators [63]. In women, both estrogens and progesterone activity lower ET-1 concentrations, and it is notably the lowest during the late follicular and luteal phase of the cycle when estradiol and progesterone levels are at their highest [64]. In anovulatory cycles there is no progesterone spike, and the luteal phase constitutes a smaller part of oligomenorrheic cycles anyway, so the compounding effect is a very big difference in estrogen/progesterone ratios between PCOS and non-PCOS women. Furthermore, one study has shown that even in the early follicular phase of eumenorrheic cycles, ET-1 is four times higher in PCOS compared to controls, and when stratifying by obesity classes the difference remains [65]. Therefore, overexpression of ET-1, in part mediated by estrogen/progesterone alterations, could be one of the mechanisms increasing CV risk in PCOS.

On the other hand, the increased level of LH, frequently found in PCOS, could have a protective effect on CV risk. The ratio between LH and follicle-stimulating hormone (FSH) concentration in the early follicular phase is normally < 1, or favoring FSH; in PCOS, inversion is often seen, with or without menstrual or ovulatory dysfunctions. While FSH receptors are specific to gonads, the LH receptor is expressed in various tissues throughout the body, blood vessels included [66]. It was demonstrated in animal and cell studies that LH/human chorionic gonadotropin (hCG) receptor activation has a biphasic vasodilating effect in vivo: The first peak is mediated by LH receptors themselves, while the following peak is mostly secondary to LH/hCG receptor-induced increase in estradiol concentration [67,68,69]. It has been shown that the main mode of direct action (the first peak) occurs by blocking the sympathetic effects on vessels, ergo independently of NO; this was also confirmed clinically in women taking exogenous βhCG therapy [70].

PCOS is characterized by menstrual cycle abnormalities and, frequently, anovulation, leading to lower monthly average progesterone levels, altered estrogen receptor activity, and increased circulating LH. However, anovulatory and/or irregular cycles are neither enough to diagnose PCOS, nor do all PCOS women have them. LH, specifically in relation to FSH (LH/FSH ratio), has been notably shown to only be elevated in less than 50% of PCOS patients [71]. Furthermore, the incidence of menstrual irregularity, as well as clinical characteristics of hyperandrogenism, fall with age [72]. Curiously, one recent Danish study of a group of women undergoing assisted fertilization has shown that even though the incidence of CV disease in PCOS was higher generally, it steadily trended lower with age, until it evened off with the control group at 50 years of age [73]. Therefore, in an attempt to evaluate CV risk in PCOS, it is plausible that the estimate of the menstrual cycle and concomitant hormonal changes need to also be considered, other than of the presence of increased LH levels.

### 3.3. Insulin Resistance

IR is the basic causal factor for type 2 diabetes mellitus (type 2 DM), and it is also connected to the metabolic syndrome, which are both risk factors for CV diseases. IR has been directly associated with chronic systemic and vascular inflammation with hypercoagulability, and finally with endothelial dysfunction, through different mechanisms [74,75]. Tissue plasminogen activator (tPA) is a membrane protease that inhibits coagulation at the interface between wall and lumen; its inhibition by plasminogen activator inhibitor 1 (PAI-1) can be increased in the milieu of IR, causing systemic hypercoagulability [76,77]. In addition, insulin binds to a G protein-coupled receptor, with PI3K/Akt and MAPK downstream pathways; those in endothelium and vascular smooth muscle cause release of NO and inhibition of endothelin. Vascular IR diminishes the activity of these pathways, negatively affecting NO production and promoting inflammatory processes. As mentioned before, ET-1 is a major factor in this regard, and there seems to be a somewhat synergistic effect with the increase in PAI-1, lowering shear stress on the wall and increasing its “stickiness” to circulating cells at the same time. Experimental data shows that here we also have selective blockage of the PI3K pathway with MAPK largely unaffected, contributing to eNOS downregulation, expression of cell adhesion molecules, and ET-1 release [78]. Moreover, hyperinsulinemia that follows IR, trying to compensate for it, has a sympathetic effect on the arteries, as well as a stimulatory effect on the renin-angiotensin system and on distal tubule reabsorption, thus decreasing natriuresis [79,80]. Together, they exacerbate vascular reactivity to other stimuli, contributing to cardiovascular dysfunction and, finally, to CV diseases.

IR is extremely frequent in PCOS. Various estimates put the prevalence in women with PCOS up to 70%, overshadowing the prevalence in general population, and the difference remains even when controlling for BMI, although obesity aggravates it [81,82]. The current understanding is that IR in PCOS is, at least in part, a consequence of hyperandrogenism, but it is a risk factor by itself; there is in fact evidence of an important interconnection between hyperandrogenism and IR in part mediated by the impact of both on adipose tissue [83,84]. There is high certainty that androgens have an effect on adipose tissue redistribution, favoring abdominal/visceral deposition instead of subcutaneous [85]. This is probably in part mediated by differential up- and downregulation of adrenergic receptors in subcutaneous and visceral fat.

Some molecular mechanisms at the basis of primary IR in PCOS have also been described. In particular, defects in efferent pathways of the insulin receptor have been shown, such as posttranslational alterations of IR components (phosphorylation mostly) that favor different MAPK pathways instead of the PI3K/Akt pathway [86]. Since inositol compounds mediate only the PI3K pathway, positive clinical data regarding the use of inositol supplementation supports the importance of this alteration [87,88,89,90]. In addition, a low expression of peroxisome proliferator agonist receptor γ (PPARγ) coactivator 1 (PGC-1) in striated muscle cells has been described, in that way interfering with mitochondrial oxidative metabolism [91,92]. Accordingly, PPARγ agonist therapy improved endothelial function in a small PCOS clinical trial [93].

There is also evidence for selective inhibition of the PI3K/Akt/mTOR pathway in visceral adipose tissue of PCOS, this time by increased expression of PI3KR1 gene, translating to the PI3K regulatory p85 subunit α [94]. Other genetic expression differences of unclear significance were also found; some of those had androgen response elements in promotor regions, which is one of the possible ways androgens could affect insulin signaling.

In either case, IR is a strong risk marker itself for CV disease and measuring it accurately would surely provide a good risk estimate in PCOS as well as in the general population. The problem with IR measurement currently is that the precise and accurate methods (hyperinsulinemic-euglycemic clamp, insulin tolerance test) are not practical, and various easy estimates (Homeostatic model assessment of insulin resistance (HOMA-IR), quantitative insulin-sensitivity check index (QUICKI), etc.) are neither precise nor accurate enough for most clinical usage [95]. A reasonable resolution to that problem would allow for IR measurement to be integrated into a comprehensive PCOS CV disease risk-assessment protocol which would include the previously mentioned parameters.

One aspect of the metabolically modifiable CV risk in PCOS is related to the endocrine disfunction of adipose tissue itself, which includes altered production of adipocytokines and a consequent impact on insulin resistance, hypertension, and prothrombotic and inflammatory processes [96,97,98]. To our best knowledge, however, this is the consequence of obesity and, particularly, of central obesity frequently present in PCOS with both normal weight and overweight/obese, and not a specific manifestation of the syndrome [8,99].

### 3.4. Low SHBG

SHBG is a liver-produced globulin, its main function being to buffer the available sex hormone concentrations in the blood. Sex hormones are bound to SHBG non-covalently, similar to albumin, but unlike albumin SHBG-bound hormones are not bioavailable. This effectively creates a control mechanism on the bioavailability of sex hormones, particularly of androgens for their high affinity to SHBG [100]. Therefore, each mechanism or substance that modifies the hepatic production of SHBG, thereby changing its own concentration in blood, finally impacts the bioavailability of sex hormones. It has been confirmed that insulin lowers SHBG concentrations in IR generally, and insulin-resistant PCOS specifically. Since SHBG normally binds T with a greater affinity than E2 [101], the downstream effect of hyperinsulinemia tends to be hyperandrogenic. Furthermore, it has been shown that in obese women SHBG affinity for T is lowered, potentially having a compound effect on IR-caused hyperandrogenism [102]. Available data implies the existence of SHBG isoforms of different chain lengths, which presumably would have differing affinities to hormones as well.

In addition, SHBG affects sex hormone activity by its direct modulation of sex hormone receptor pathways. Most of the research on this topic was motivated by the inquiry into a significantly higher incidence of autoimmune diseases in women, resulting in findings currently best explained by SHBG having an active membrane receptor [103]. There are several candidates for an SHBG receptor, one well-studied option being megalin [104]. Megalin is a membrane protein that binds to SHBG and internalizes it, together with the hormones attached to it. There is also evidence of cAMP spikes following the binding of SHBG to its receptor, resulting in increased cellular activity—the exact effect depends on the cell line [105]. It is as of yet unclear how important this complex endocytosis mechanism is for sex hormone activity. It is now known that SHBG is also produced in sex hormone-responsive tissues like the placenta, testis, brain, and T lymphocytes [106]; this could have major consequences on the hormone activity locally without being noticeable by venous blood analysis. Furthermore, there is also evidence of androgens themselves affecting the concentration of SHBG: One meta-analysis on androgenic therapy trials in post-menopausal women has shown that exogenous T therapy in post-menopausal women lowers SHBG [107], thus suggesting a possible vicious circle in hyperandrogenic women.

When talking about CV risk, SHBG is also involved in mechanisms not related to sex hormones. The SHBG gene has an unusual promotor region with a variable (TAAAA)n repeating sequence, which affects its transcriptional activity—lower or higher than optimal 7–8 repeats are related to lower production of SHBG [108,109]. This polymorphism was shown to correlate to CV risk factors like cIMT and flow-mediated dilation (FMD) [110]. Other mutations in the coding sequence are directly predictive of the development of type 2 DM, some increasing risk, others being protective of type 2 DM. Genetic data were also used in a mendelian randomization analysis, which suggests that low levels of SHBG could be involved in type 2 DM pathogenesis. Epidemiological studies like the large SWAN longitudinal study have confirmed that low SHBG is a predictor of CV disease [111,112,113].

In PCOS, SHBG is generally lower than in non-PCOS women of similar age. Although its activity is heavily influenced by insulin and sex hormones, there is enough data to point to it being independently considered in the previously hypothesized PCOS CV disease risk calculation. One great advantage of SHBG in this regard is the relative stability of its concentration in the blood, as well as ease of measuring it. This notwithstanding, more research in this area is certainly necessary.

## 4. Summary and Conclusions

**Figure 1:**
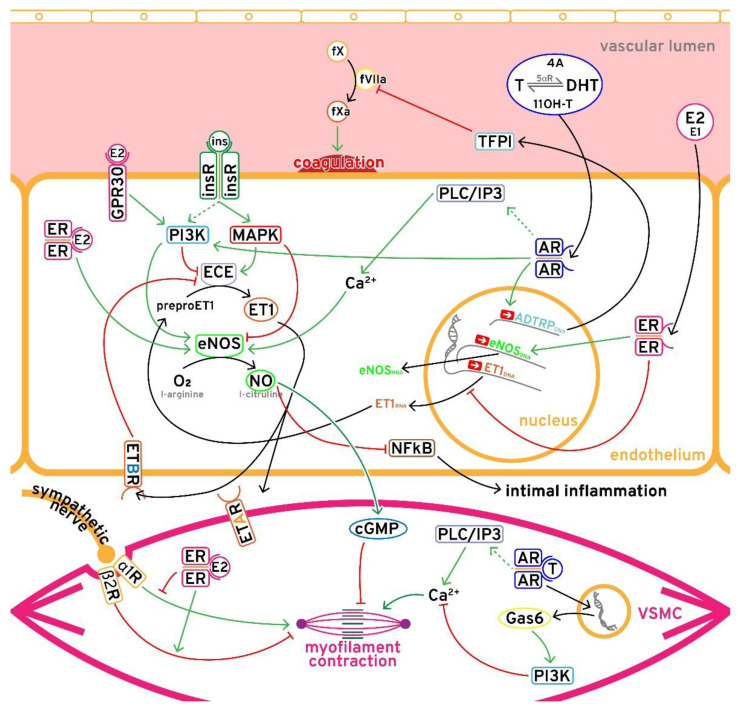
Complex interaction between the sex hormones, insulin, and vascular regulatory mechanisms in the endothelium and vascular smooth muscle cells (VSMC). fX: coagulation factor X; fVIIa: activated coagulation factor VII; 4A: androstenedione; T: testosterone; 5αR: 5-alpha-reductase; DHT: dihydrotestosterone; 11OH-T: 11-hydroxytestosterone; E2: estradiol; E1: estrone; GPR30: G-protein coupled membrane receptor 30; ins: insulin; insR: insulin receptor; PLC/IP3: phospholipase C/inositol 1,4,5-triphosphate; PI3K: phosphoinositide 3-kinase; MAPK: mitogen-activated protein kinase; AR: androgen receptor; ECE: endothelin-converting enzyme; ET1: endothelin-1; eNOS: endothelial nitric oxide synthase; NO: nitric oxide; ADTRPDNA: androgen-dependent TFPI-regulating protein gene; eNOSDNA: eNOS gene; ET1DNA: preproET1 gene; ER: estrogen receptor; NFκB: nuclear factor kappa-light-chain-enhancer of activated B cells; ETAR: endothelin A receptor; ETBR: endothelin B receptor; cGMP: cyclic guanosine monophosphate; α1R: α-1 adrenergic receptor; β2R: β-2 adrenergic receptor; Gas6: growth arrest-specific gene 6; VSMC: vascular smooth muscle cell.

This review tried to sketch out the state of this multidisciplinary topic, and more importantly, point out the “rough edges” in our fundamental understanding of CV risk in PCOS. With that said, it seems that the complexity of clinical data regarding CV risk in PCOS is very much a reflection of complexity at the molecular level (Figure 1), and of the heterogenicity of the syndrome. On the CV risk side, we have many biochemical markers (hyperandrogenism, estrogen/progesterone imbalance, IR, compensatory hyperinsulinemia, and low SHBG) which tell us that PCOS should be regarded as a high-risk state, while minimal CV event data show us inconclusive results. The non-correspondence of alterations of biomediators of CV risk, early atherosclerotic processes, and CV events in PCOS later in life could be a product of lacking clinical data (prospective longitudinal studies) with adequate cohorts that are followed long enough, the extreme heterogenicity of the cohorts of PCOS included in the studies, or of their inadequate phenotypization in terms of biomediators of CV risk, in part due to the inadequacy of the methods used to measure it. In the meantime, the understanding of the underlying biochemical processes of CV risk in PCOS shifts attention to specific subgroups of the PCOS population which potentially carry most of the risk for CV events.
